# Serial T1 mapping of right ventricle in pulmonary hypertension: comparison with histology in an animal study

**DOI:** 10.1186/s12968-021-00755-y

**Published:** 2021-05-27

**Authors:** Pan Ki Kim, Yoo Jin Hong, Hyo Sub Shim, Dong Jin Im, Young Joo Suh, Kye Ho Lee, Jin Hur, Young Jin Kim, Byoung Wook Choi, Hye-Jeong Lee

**Affiliations:** 1 Department of Radiology, Research Institute of Radiological Science, Severance Hospital, Yonsei University College of Medicine, 50-1 Yonsei-ro, Seodaemun-gu, Seoul, 03722 South Korea; 2 Department of Pathology, Severance Hospital, Yonsei University College of Medicine, 50-1 Yonsei-ro, Seodaemun-gu, Seoul, 03722 South Korea

**Keywords:** Pulmonary hypertension, Right ventricular remodeling, Myocardial fibrosis, T1 mapping technique, Extracellular volume fraction

## Abstract

**Background:**

Right ventricular (RV) free wall fibrosis is an important component of adverse remodeling with RV dysfunction in pulmonary hypertension (PH). However, no previous reports have compared cardiovascular magnetic resonance (CMR) findings and histological analysis for RV free wall fibrosis in PH. We aimed to assess the feasibility of CMR T1 mapping with extracellular volume fraction (ECV) for evaluating the progression of RV free wall fibrosis in PH, and compared imaging findings to histological collagen density through an animal study.

**Methods:**

Among 42 6-week-old Wistar male rats, 30 were classified according to disease duration (baseline before monocrotaline injection, and 2, 4, 6 and 8 weeks after injection) and 12 were used to control for aging (4 and 8 weeks after the baseline). We obtained pre and post-contrast T1 maps for native T1 and ECV of RV and left ventricular (LV) free wall for six animals in each disease-duration group. Collagen density of RV free wall was calculated with Masson's trichrome staining. The Kruskall-Wallis test was performed to compare the groups. Native T1 and ECV to collagen density were analyzed with Spearman’s correlation.

**Results:**

The mean values of native T1, ECV and collagen density of the RV free wall at baseline were 1541 ± 33 ms, 17.2 ± 1.3%, and 4.7 ± 0.5%, respectively. The values of RV free wall did not differ according to aging (*P* = 0.244, 0.504 and 0.331, respectively). However, the values significantly increased according to disease duration (*P* < 0.001 for all). Significant correlations were observed between native T1 and collagen density (*r* = 0.770, P < 0.001), and between ECV and collagen density for the RV free wall (*r* = 0.815, P < 0.001) in PH. However, there was no significant difference in native T1 and ECV values for the LV free wall according to the disease duration from the baseline (*P* = 0.349 and 0.240, respectively).

**Conclusions:**

We observed significantly increased values for native T1 and ECV of the RV free wall without significant increase of the LV free wall according to the disease duration of PH, and findings were well correlated with histological collagen density.

**Supplementary Information:**

The online version contains supplementary material available at 10.1186/s12968-021-00755-y.

## Background

In pulmonary hypertension (PH), the right ventricle (RV) is compromised by high pressure, which leads to RV cavity dilatation, dysfunction, and fibrosis. Because RV performance is an indicator of disease severity and a predictor of survival in PH, there is great interest in new imaging techniques that allow early detection of RV involvement [1–3]. Although echocardiography provides some information on RV structure and function [4], cardiovascular magnetic resonance (CMR) has become the gold standard in this regard due to the structural complexity of the RV [5]. However, until now, the greatest advantage of CMR for the RV has been its high-resolution cine imaging rather than contrast-enhanced myocardial fibrosis imaging.

While left ventricular (LV) fibrosis is readily evaluated with CMR using late gadolinium enhancement (LGE) imaging and T1 mapping with the extracellular volume fraction (ECV) [6–8], there are technical difficulties when assessing RV free wall fibrosis because of insufficient spatial resolution due to the thinness of the RV wall. Hence, the assessment of myocardial fibrosis in PH has been limited to ventricular septal insertion points (SIPs) rather than the RV free wall [9–11]. However, because RV free wall fibrosis is also a significant feature of PH, it should be regarded as an important component of adverse remodeling with RV dysfunction [12]. Recently, advances in CMR native T1 mapping have enabled higher spatial resolution and shown increases in native T1 and ECV for the RV free wall in PH patients, with these findings being associated with decreased RV function [13–15]. Moreover, T1 mapping with ECV might make it feasible to evaluate gradual changes in diffuse interstitial fibrosis in the overloaded RV of PH in contrast to LGE imaging, which can primarily detect focal areas of fibrosis with its dichotomous nature. However, no reports have yet compared native T1 mapping with ECV and histological analysis for tissue characterization of the RV free wall in PH. Therefore, we aimed to assess whether it was possible to use T1 mapping with ECV to evaluate the sequential progression of RV free wall fibrosis in PH, and compare the CMR findings to histological collagen density with a translational animal model.

## Methods

### Animal model

All experiments were approved by the Animal Care and Use Committee of our institution, and we performed experiments according to the National Institutes of Health guidelines. A total of 46 6-week-old Wistar male rats were used. A single subcutaneous injection of monocrotaline (40 mg/kg) (Sigma Chemical, St. Louis, Missouri, USA) was used to induce PH [16, 17]. Animals were divided into 5 groups according to disease duration; baseline before injection, and 2, 4, 6 and 8 weeks after injection, respectively. Among 46 animals, 4 expired before the study was completed (1 at 4 weeks, 1 at 6 weeks, and 2 at 8 weeks), and 6 animals allocated to each of the 5 disease-duration groups. The remaining 12 6-week-old Wistar male rats were used to control for confounding factors thought to arise from aging, and 6 animals were assigned to each group after 4 weeks and 8 weeks after the baseline time point without monocrotaline injection. The weight of each animal was measured immediately before CMR scanning to calculate body surface area [18]. Venous blood samples were obtained from the tail vein for hematocrit levels to calculate ECV.

### Cardiovascular magnetic resonance imaging

All CMR images were acquired through a 9.4 T small animal CMR scanner (Bruker Biospin, Billerica, Massachusetts, USA) with an 86-mm volume transmit coil and a 4-channel phased-array surface coil. CMR was performed under spontaneous ventilation with anesthesia by inhalation of 2% isoflurane. Cine images in the short-axis planes were acquired using a self-gated fast low-angle shot sequence (IntraGate, Bruker BioSpin, ParaVision 5.1) with the following parameters: TR/TE, 5/2.3 ms; flip angle, 10°; field of view, 40 × 40 mm; pixel size, 0.32 × 0.32 mm^2^; section thickness, 2 mm; number of slice, 8—9; and cardiac phase, 25. Pre-contrast T1 maps were acquired in the short axis planes using a respiratory navigated Look-Locker inversion recovery imaging [19] (Additional file 1: Fig. S1). The sequence was proposed to provide higher motion robustness so that the myocardial T1 maps could be accurately measured in small animals with higher heart rates and respiratory rates. The sequence is based on the Look-Locker sequence, which continuously acquires with a spoiled gradient-echo readout sequence during T1 recovery following the inversion pulse (IR) without respiratory gating. Therefore, the sequence is more suitable for small animals with heart rates of 300–400 bpm. In this T1 mapping sequence, image data was acquired consecutively during T1 recovery process with electrocardiographic (ECG) gating. Respiratory motion compensated IR-weighted images were reconstruced using a self-gating method that estimates respiratory motion from the acquired image data itself [20]. Scanning parameters were as follows: TR/TE, 15/1.52 ms, flip angle, 8°; field of view, 40 × 40 mm, pixel size = 0.2 × 0.2 mm^2^; section thickness, 1.5 mm; slice gap, 1.5 mm; acquisition duration, 16 heart beats; number of slice, 3; and relaxation duration, 5000 ms. The RV myocardial thickness of normal Wistar male rats was approximately 0.6 mm in diastole and 0.9 mm in systole. The relative spatial resolution was 0.2 × 0.2 mm^2^. Post-contrast T1 maps were obtained with the same protocol 15 min after administration of the contrast agent (gadoterate meglumine, 0.2 mmol/kg; Dotarem; Guerbet, Roissy, France). T1 maps were generated by applying three-parameter non-linear least square fitting on a pixel-by-pixel basis for the motion-corrected phase-sensitive inversion recovery images and then Look-Locker correction [21].

**Fig. 1:**
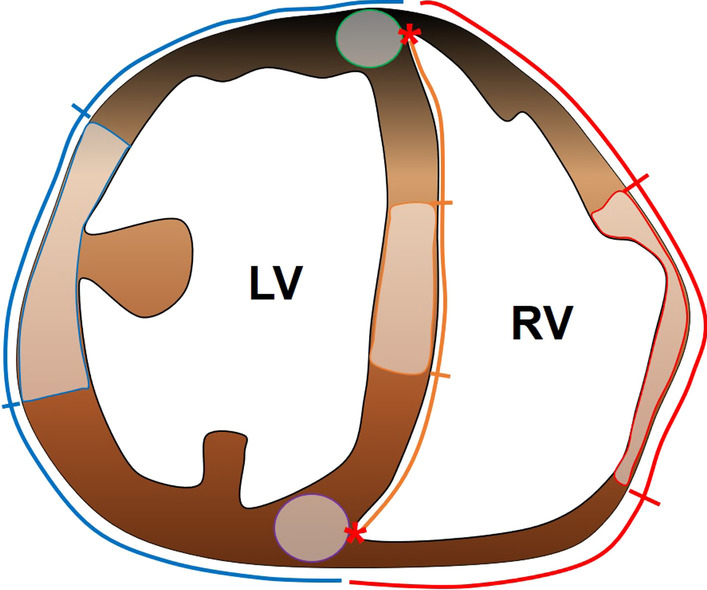
A diagram to measure T1 values on the myocardium. Each region-of-interest (ROI) was drawn on the middle third of the free walls of both ventricles (right ventricle (RV) outlined in red and left ventricle (LV) outlined in blue) and septum (orange line) based on the inner junctional points (red asterisks) in reference to the exact middle of the LV on the short axis plane. Additionally, circular ROIs were drawn at the anterior (green circle) and inferior (purple circle) septal insertion points (SIPs)

All CMR images were evaluated with commercially available software (cvi42; Circle Cardiovascular Imaging, Calgary, Alberta, Canada). Two radiologists (Y.J.H. and H-J.L. with 10 and 12 years of experience in cardiovascular imaging) reviewed the images in consensus and were blinded to disease duration. From the short-axis cine images, the end-diastolic and systolic volumes of both ventricles were calculated by multiplying the areas enclosed by the endocardial contours by slice thickness. The end-diastolic volume indices (EDVI) were calculated by dividing the end-diastolic volume by body surface area (m^2^). The ejection fraction (EF) was calculated with the following equation: (end-diastolic volume − end-systolic volume) × 100/end-diastolic volume (%). The ECV was measured using pre- and post-contrast T1 maps as follows: (ΔR1_m_/ΔR1_b_) × (1 − hematocrit) × 100 (%), for which R1_m_ is R1 in the myocardium, R1_b_ is R1 in the blood, and ΔR1 is the change in T1 relaxivity before and after the contrast agent was administered. T1 measurements were performed on the middle third of the RV free wall, septum and LV lateral wall right at the midway between the apex and base of the LV on short axis plane. When measuring the middle third of RV free wall, the inner junctional points of both ventricles were used as positional reference points to match the imaging findings with the histologic findings (Fig. 1). The endo- and epicardial borders of the myocardium were delineated manually for T1 measurements, and the very edges were excluded to reduce partial volume averaging from the myocardial-blood interface. Additionally, T1 measurements were done at the anterior and inferior SIPs with circular regions of interest (ROI) of approximately 2 mm^2^. Blood T1 values were also measured in the LV cavity while avoiding papillary muscles.

### Pathology

After CMR imaging, all hearts were resected and fixed in 4% formaldehyde, and sliced in sections approximately 2-mm thick right at the midway between the apex and base of the LV on the short-axis plane. The sections were embedded in paraffin and were sliced further into 5-µm-thick sections. Afterwards, the specimens were stained with H&E and Masson’s trichrome staining. Histologic analysis was performed by a pathologist H.S.S. with 13 years of experience in thoracic pathology blinded to disease duration and CMR findings. H&E-stained sections were used for the overall examination. Masson’s trichrome-stained sections were digitally scanned with high-power magnification (400X) (ImageScope v10.0; Aperio Technologies, Vista, California, USA), and the pixel numbers of the myocardial tissue and collagen were counted using a macro written in ImageJ (National Institute of Health, Bethesda, Maryland, USA) based on the thresholding algorithms [22, 23]. Collagen density for the RV was calculated as the percentage of pixel numbers of collagen to the myocardial tissue within a defined ROI, which was the same on T1 maps located at the middle one third level of the RV free wall.

### Statistical analyses

All statistical analyses were performed with commercially available software SPSS Statistics (version 20.0, Statistical Package for the Social Sciences, International Business Machines, Inc., Armonk, New York, USA). Continuous variables are expressed as means ± standard deviations using 2 significant figures, and the Kruskall-Wallis test was performed to estimate significant differences among the groups (from baseline to 8 weeks) for continuous variables. Then for variables with significant differences on the Kruskall-Wallis test, the Mann–Whitney U test was further performed to analyze the differences between the baseline and each disease-duration group, and the differences between two sequentially adjacent disease-duration groups. Because our study was based on an exploratory data analysis with animal experiments, we did not use the Bonferroni correction for multiple testing. Correlations of native T1 and ECV to collagen density of the RV free wall were analyzed with Spearman’s correlation. A *P* value < 0.05 was considered statistically significant.

## Results

### Baseline characteristics

Baseline characters are summarized in Table 1. The mean baseline body weight was 267.8 ± 8.1 g, and body weight increased according to age and disease duration (*P* < 0.001). The mean hematocrit level for ECV calculations was 53.8 ± 2.0% at baseline, and increased according to age and disease duration (*P* < 0.001), with 6 weeks and 8 weeks showing significantly increased levels compared to the baseline (*P* = 0.004 and 0.002, respectively). The mean heart rate was not significantly different according to age and disease duration (*P* = 0.648).

**Table 1:** Results of basic characteristics according to disease duration

	Baseline	2 weeks	4 weeks	6 weeks	8 weeks
Body weight (g)	267 ± 8	311 ± 9	343 ± 7	369 ± 12	418 ± 8
*P* value*		0.002	0.002	0.002	0.002
*P* value^†^		0.002	0.002	0.002	0.002
Hematocrit (%)	53.8 ± 2.0	54.1 ± 1.6	57.0 ± 3.0	60.1 ± 2.7	62.0 ± 2.8
*P* value*		0.729	0.071	0.004	0.002
*P* value^†^		0.729	0.097	0.132	0.310
Heart rate (bpm)^‡^	283 ± 13	282 ± 16	285 ± 15	289 ± 13	296 ± 16

Data are means ± standard deviations

*Between the baseline group and each duration group

^†^Between two sequentially adjacent duration groups

^‡^No significant difference seen with the Kruskall–Wallis test (*P* = 0.648)

### Cardiac function

**Fig. 2:**
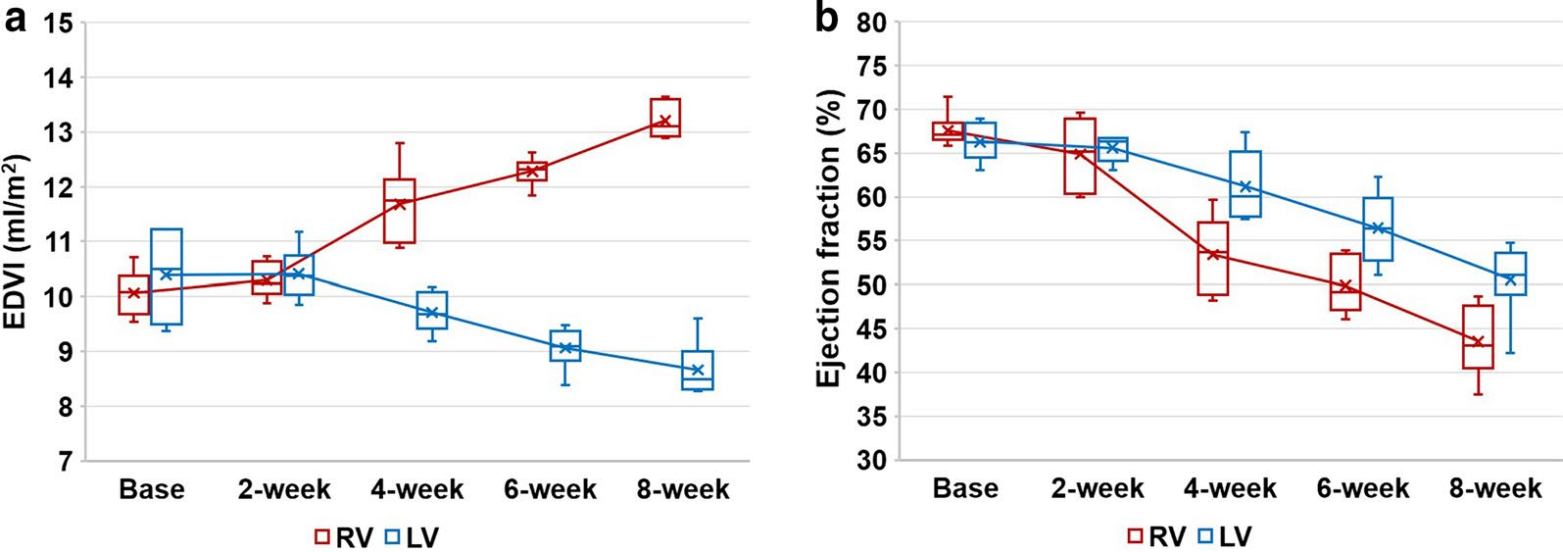
Sequential progression of cardiac function according to disease duration. Both ventricular end-diastolic volume indices (EDVI) (**a**) and both ventricular ejection fractions (EF) (**b**) show significant changes according to disease duration

The results of cardiac function are described in Fig. 2 and Additional file 1: Table S1. The mean baseline values of RVEDVI and LVEDVI were 10.1 ± 0.4 ml/m^2^ and 10.4 ± 0.9 ml/m^2^, and of RVEF and LVEF were 67.6 ± 2.0% and 66.3 ± 2.2%, respectively. The mean values of RVEDVI increased and the mean values of LVEDVI decreased according to disease duration (*P* < 0.001 for both) (Fig. 2a). The mean values of RVEF and LVEF decreased according to disease duration (*P* < 0.001 for both) (Fig. 2b). For RVEDVI and RVEF, significant differences were first observed at 4 weeks compared to the baseline (*P* = 0.004 for both), and the values did not show significant differences between 4 and 6 weeks (*P* = 0.109 and 0.150, respectively); However, significant differences were observed between 6 and 8 weeks (*P* = 0.004 and 0.025). For LVEDVI, significant differences were observed at 6 weeks compared to the baseline (*P* = 0.004), and no significant differences were observed between 6 and 8 weeks (*P* = 0.180). For LVEF, significant differences were first observed at 4 weeks compared to the baseline (*P* = 0.037). In addition, no significant differences were observed between 4 and 6 weeks (*P* = 0.055), but a significant difference was observed between 6 and 8 weeks (*P* = 0.030).

### Myocardial native T1 and ECV

**Fig. 3:**
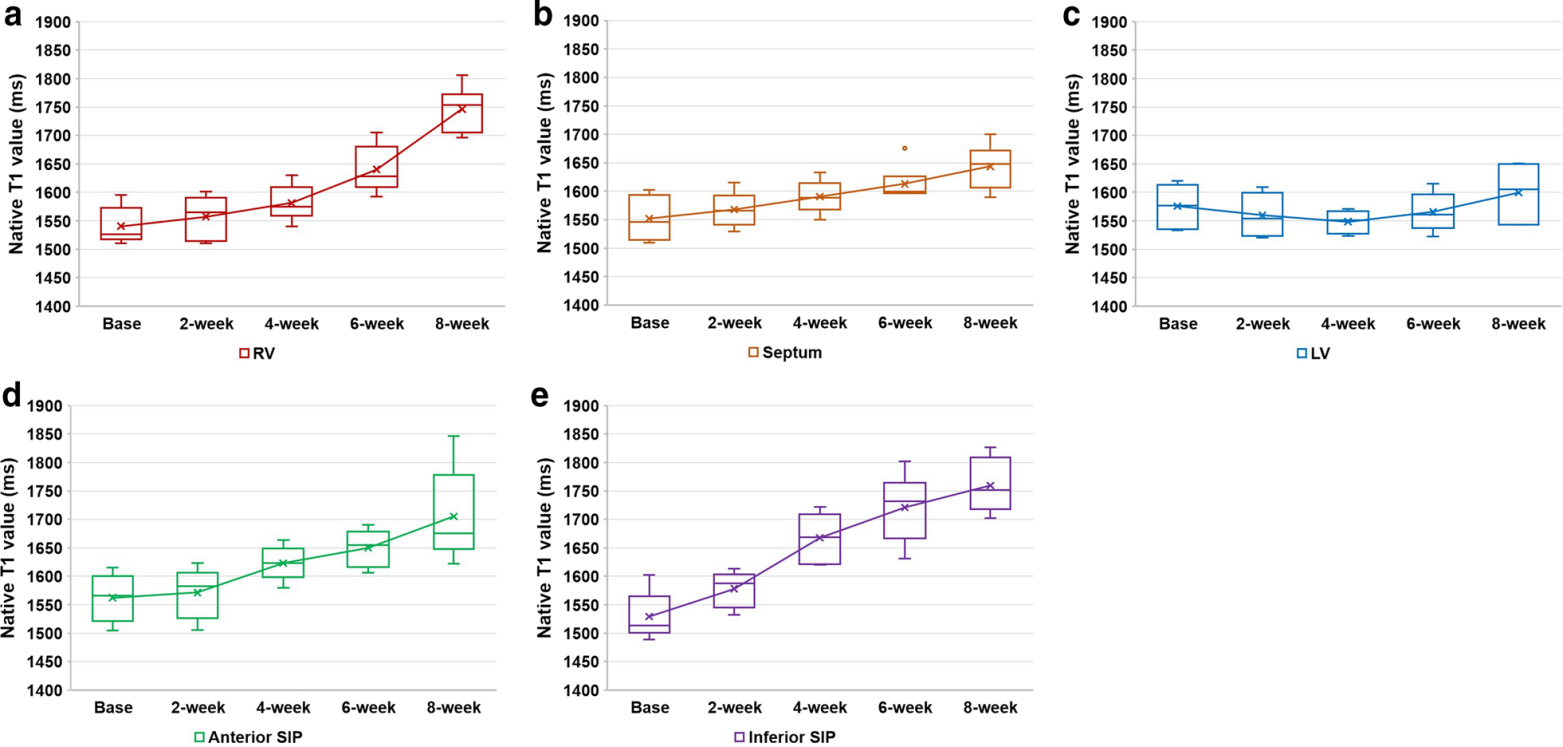
Sequential progression of myocardial native T1 according to disease duration. The mean values of native T1 for RV (**a**), septum (**b**), anterior SIP (**c**) and inferior SIP (**d**) significantly increase according to disease duration except for the LV (**e**)

The results of myocardial native T1 are described in Fig. 3 and Additional file 1: Table S2. The mean values of native T1 on baseline group were 1541 ± 33 ms, 1552 ± 39 ms, 1562 ± 42 ms, 1529 ± 42 ms, and 1576 ± 37 ms for the RV, septum, anterior SIP, inferior SIP, and LV, respectively. The mean values of native T1 for each section significantly increased according to disease duration (*P* < 0.001 for the RV and inferior SIP, 0.006 for the septum, and 0.001 for the anterior SIP, respectively) except for the LV (*P* = 0.349). Significant differences were first observed at 6 weeks for the RV and septum (*P* = 0.004 and 0.022, respectively), and further significant differences were observed between 6 and 8 weeks (*P* = 0.004) for the RV, but not for the septum (*P* = 0.223). For the anterior SIP and inferior SIP, the first significant difference was observed at 4 weeks (*P* = 0.026 and 0.002, respectively), but no further significant differences were observed between 4 and 6 weeks (*P* = 0.180 and 0.132, respectively), and between 6 and 8 weeks (*P* = 0.240 and 0.310, respectively).

**Fig. 4:**
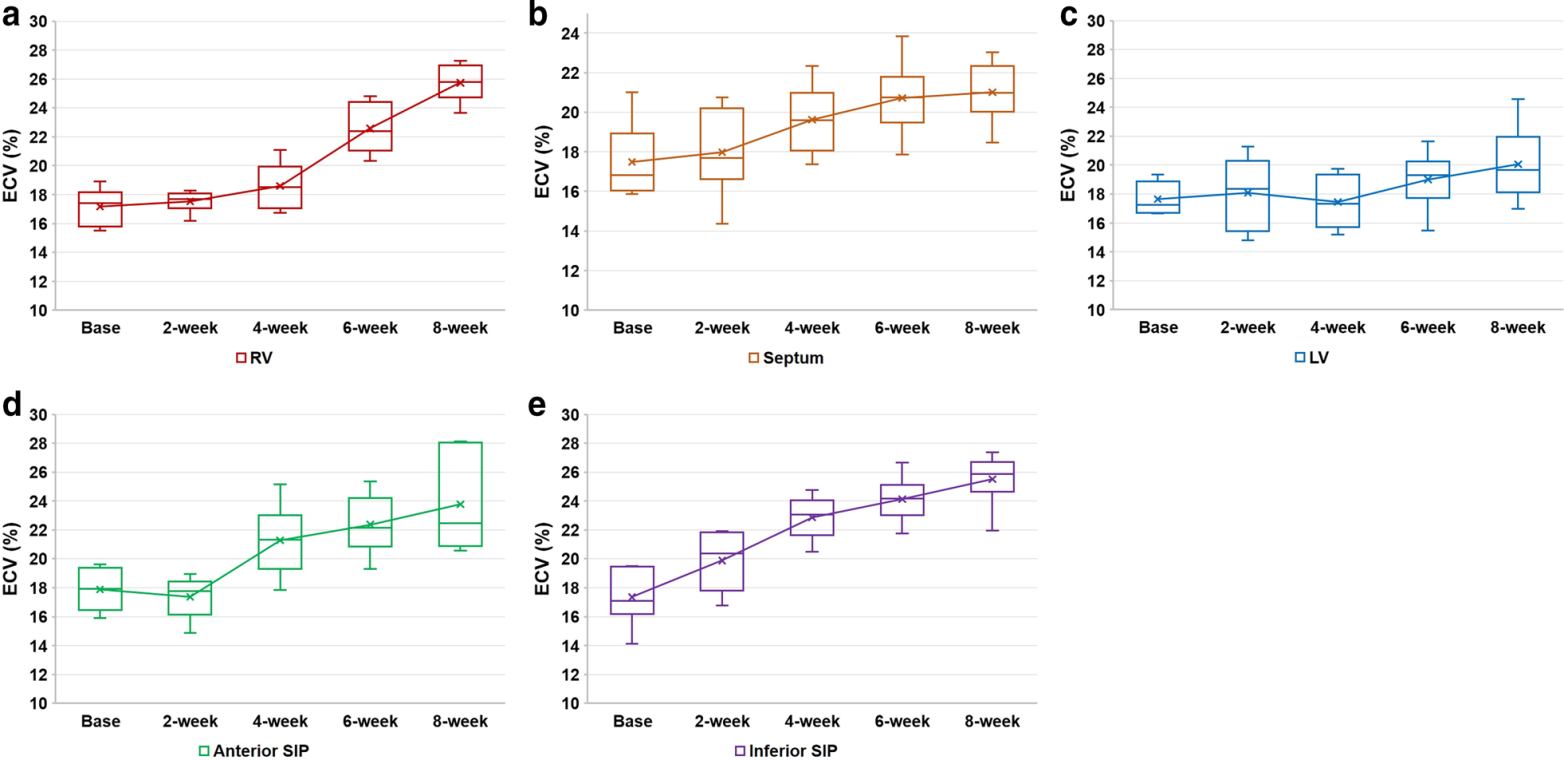
Sequential progression of myocardial ECV values according to disease duration. The mean values of extracellular volume fraction (ECV) for RV (**a**), septum (**b**), anterior SIP (**c**) and inferior SIP (**d**) significantly increase according to disease duration except for the LV (**e**)

The results of ECV are described in Fig. 4 and Additional file 1: Table S3, which showed similar patterns to those of native T1. The mean values of ECV on baseline group were 17.2 ± 1.3%, 17.5 ± 2.0%, 17.9 ± 1.5%, 17.4 ± 2.0%, and 17.7 ± 1.1% for the RV, septum, anterior SIP, inferior SIP, and LV, respectively. These mean values of ECV showed significant increase according to disease duration (*P* < 0.001 for the RV and inferior SIP, 0.020 for the septum, and 0.001 for the anterior SIP, respectively) except for the LV (*P* = 0.240). The first significant difference compared to the baseline was observed at 6 weeks for the RV and septum (*P* = 0.002 and 0.041, respectively). Additional significant differences between 6 and 8 weeks were observed for the RV (*P* = 0.009), but not for the septum (*P* = 0.818). For the mean ECV values of the anterior SIP and inferior SIP, significant differences were first observed at 4 weeks compared to the baseline (*P* = 0.015 and 0.002, respectively), but with no significant differences between 4 and 6 weeks (*P* = 0.485 and 0.240, respectively), and between 6 and 8 weeks (*P* = 0.818 and 0.180, respectively).

### Histopathology

**Fig. 5:**
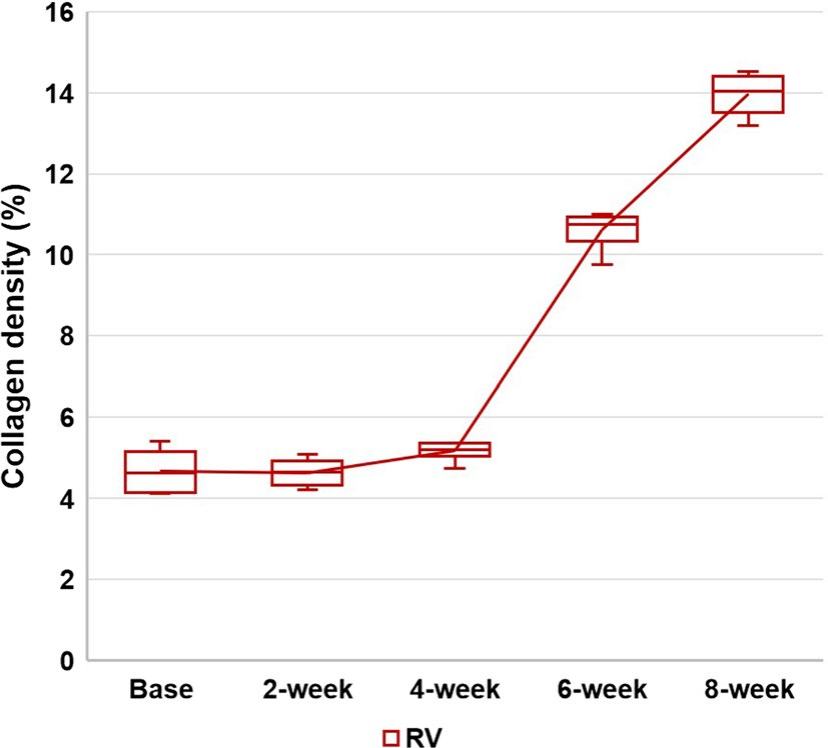
Sequential progression of collagen density in the RV free wall according to disease duration. The mean values of collagen density significantly increase according to disease duration

**Fig. 6:**
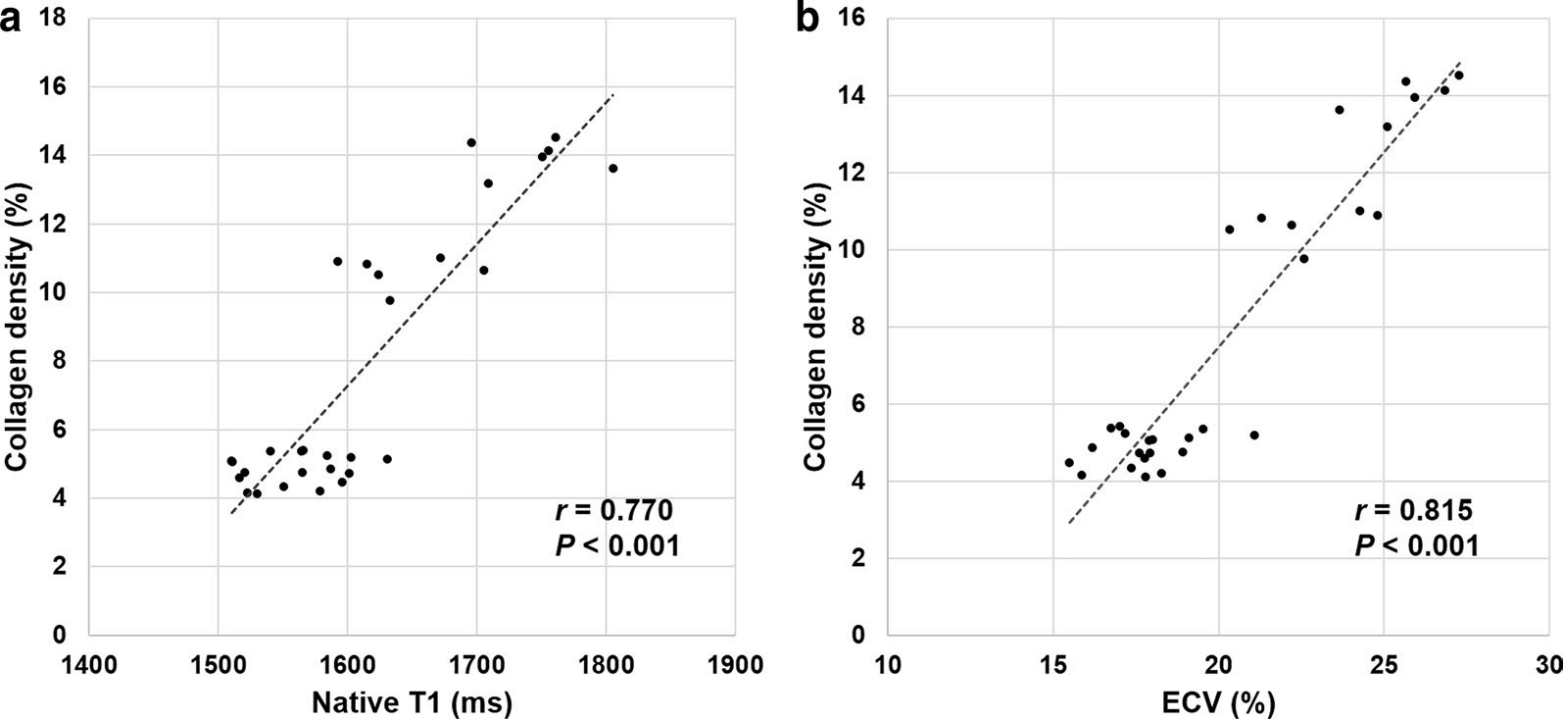
Correlations of native T1 and ECV to collagen density of the RV free wall. Significant correlations were observed between native T1 and collagen density, and between ECV and collagen density for the RV free wall

**Fig. 7:**
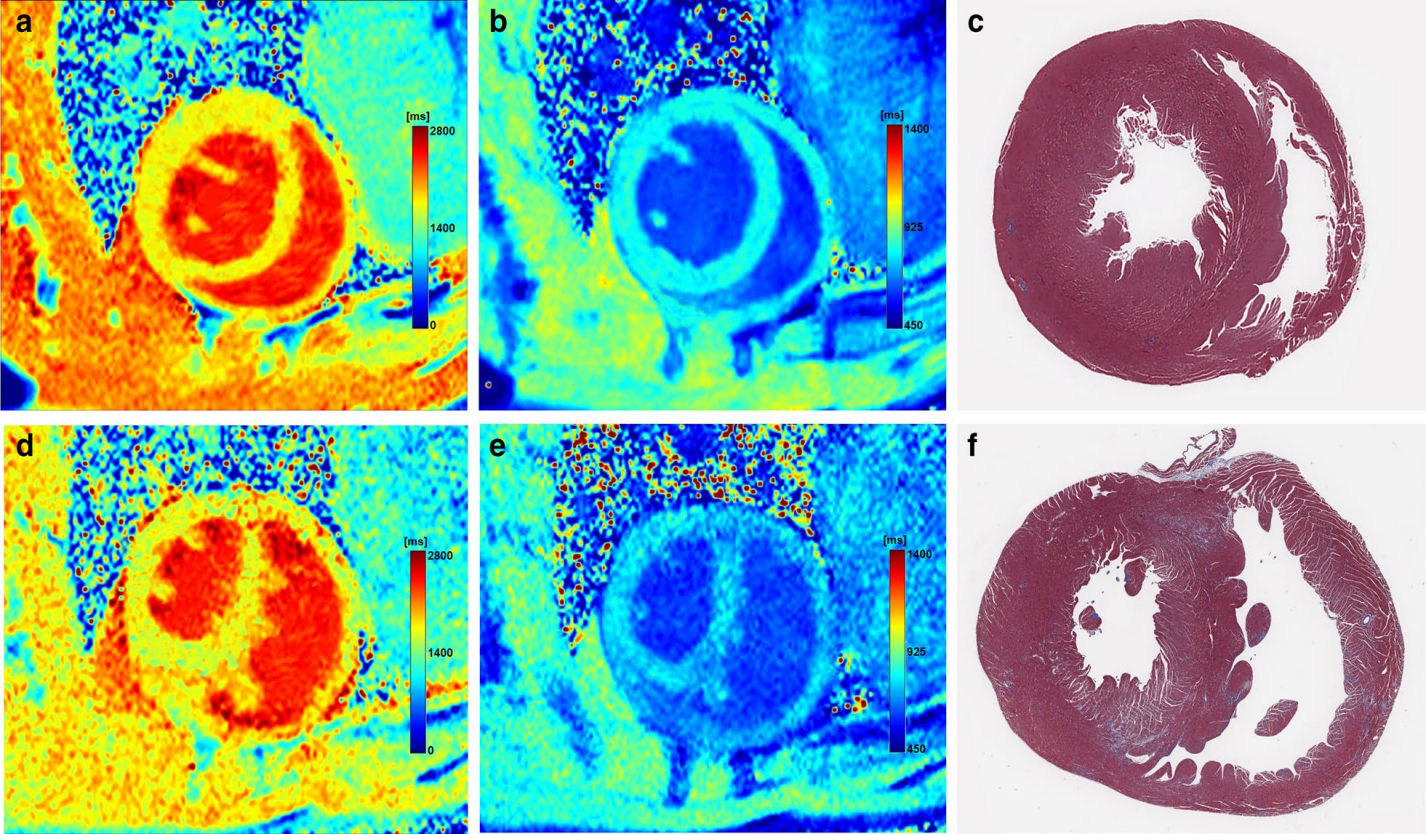
Representative cases from our study. Myocardial native T1 and ECV values of the RV free wall are 1564 ms and 17.8% from the pre- and post-contrast images (**a**, **b**), and collagen density is 4.1% on myocardial specimens at baseline (**c**). Pre- and post-contrast images obtained 6 weeks after monocrotaline injection (**d**, **e**) show increased native T1 and ECV values for the RV free wall (1672 ms and 24.3%, respectively). Collagen density is 11.0% (**f**). Myocardial specimens refer to myocardial specimens scanned digitally with Masson’s trichrome staining (original magnification × 400)

Animals showed varying degrees of RV hypertrophy macroscopically dependent on disease duration. Microscopic analysis found evidence of varying degrees of myocyte hypertrophy, interstitial fibrosis, muscular fiber disarray, and lymphocytic infiltration with collagen deposition according to disease duration. The mean values of collagen density for the RV were 4.7 ± 0.5%, 4.6 ± 0.3%, 5.2 ± 0.2%, 10.6 ± 0.5%, and 14.0 ± 0.5% at the baseline, 2 weeks, 4 weeks, 6 weeks, and 8 weeks, respectively (Fig. 5). The values significantly increased with disease duration (*P* < 0.001) and showed similar patterns with native T1 and ECV of the RV free wall. The first significant increase was observed at 6 weeks (*P* = 0.002) and another significant increase was observed between 6 and 8 weeks (*P* = 0.002). In addition, significant correlations were observed between native T1 and collagen density (*r* = 0.770, P < 0.001), and between ECV and collagen density for the RV free wall (*r* = 0.815, P < 0.001). (Fig. 6 and Fig. 7).

### Normal control study

The results of the normal control study are described in Additional file 1: Table S4. Mean body weight increased with age (*P* < 0.001). However, there were no significant differences for mean hematocrit levels and heart rates (*P* = 0.264 and 0.076, respectively). For cardiac function, there were no significant differences between RVEDVI and LVEDVI (*P* = 0.244 and 0.147, respectively) as well as between RVEF and LVEF (*P* = 0.171 and 0.580, respectively). When characterizing myocardial tissue, native T1 values (*P* = 0.244 and 0.548 for RV and LV, respectively) and ECV values (*P* = 0.504 and 0.291 for RV and LV, respectively) of both ventricles showed no significant difference at baseline, 4 weeks, and 8 weeks. In addition, no significant differences were observed for collagen density in the RV according to age (*P* = 0.331).

## Discussion

The major finding of our study was the significant increase observed in native T1 and ECV values of the RV free wall without significant increases in the LV free wall according to disease duration, and the results were well correlated with collagen density on histopathological analysis. In addition, similar patterns of increase were observed in native T1, ECV and collagen density of the RV free wall with first significance at 6 weeks compared to the baseline and then further significance between 6 and 8 weeks. Through the normal control study, we found that no significant difference was observed in the native T1 and ECV values of both ventricles according to age. T1 mapping with ECV has been associated with diffuse myocardial fibrosis or infiltrates of LV through histological validation [6–8]. In addition, an animal study validated the use of T1 mapping with ECV for the serial monitoring of chemotherapy-induced cardiotoxicity [24]. However, there have been no reports on the RV free wall. Therefore, our study results suggest that T1 mapping with ECV can provide important insight into myocardial tissue characteristics and indicate increased fibrosis in the RV free wall through histologic comparison.

Furthermore, we observed significant increase in native T1 and ECV values of the interventricular septum including SIPs as well as the RV free wall according to disease duration in animal models. Interestingly, these increases in the values of each section fluctuated from time to time, rather than gradually increasing linearly with time. For the anterior SIP and inferior SIP, significant increase in fibrosis was detected relatively early at 4 weeks with significant changes in both ventricular functions, and thereafter, no further significant changes were observed. Both ventricular functions did not show significant changes between 4 and 6 weeks, but did show significant changes between 6 and 8 weeks. In contrast, significant increases in fibrosis were observed for the RV and septum relatively late at 6 weeks, and further significant progression was noted for the RV at 8 weeks. From these findings, we hypothesized that early changes in the RV function were due to exposure to increased afterload, and that mechanical stress associated with structural changes of the paradoxical septal motion caused fibrosis in the SIPs [25]. Afterwards, continuous increases in the afterload to the RV triggered inflammation, which in turn progressed to RV free wall fibrosis, and eventually exacerbated RV dysfunction [12].

While prior studies showed modest increase in the fibrosis of the RV free wall in human PH patients than in controls [26, 27], we noted obviously increased fibrosis in the RV free wall in the later stages of PH compared to the baseline. The timing of the tissue sampling might explain these differences because we observed more severe fibrosis in the RV free wall later in the disease. In addition, we used monocrotaline to induce PH in the study animals. This is a simple and reliable method to induce PH in animal models and its results resemble the vasculopathy of human pulmonary arterial hypertension [16, 17]. However, monocrotaline-induced PH shows rapid disease progression over several weeks in contrast to human PH, which evolves slowly over years. Therefore, the process of myocardial fibrosis might be different for animals and humans. Further, monocrotaline could induce myocarditis in animals. However, while the induction dose of myocarditis is known to be 50-60 mg/kg, we used 40 mg/kg in the study. In addition, significant increases in myocardial native T1 and ECV values were not observed in the LV according to disease duration. Therefore, direct myocardial injury from monocrotaline might be insignificant in the present study.

### Limitations

Our study has limitations. First, we did not perform a longitudinal observation because frequent administration of anesthesia for CMR scanning often leads to the death of animals with PH, and that would have impeded the progress of our study. However, because the effect of monocrotaline has been reported to be constant throughout time in previous reports [16], the serial changes observed in this study might be acceptable. Second, a variety of different histological methods are currently used to quantify collagen in myocardial fibrosis, and the amount of estimated collagen density can vary slightly depending on the quantification method [28]. Third, we measured native T1 and ECV values with CMR and collagen density with histologic specimens in the middle one third level of the RV to match the corresponding locations. However, slight differences might have been present.

## Conclusions

We observed the sequential progression of RV free wall fibrosis with time to time variations in an animal model with PH using CMR native T1 mapping and ECV; findings well correlated with histological analysis. As described previously, RV free wall fibrosis is a highly dynamic process, which can be reversed after lung transplantation or pulmonary endarterectomy. Therefore, further studies are needed to determine whether the T1 mapping technique can be used to evaluate the reversibility of RV free wall fibrosis. In addition, the T1 mapping technique needs to be developed further for humans with larger scales of investigation before we can determine the clinical value of the T1 mapping technique for RV free wall fibrosis. New technical developments and subsequent findings might provide new perspectives on PH and help clinicians develop more management strategies for PH patients in the future.

## Supplementary Information

**Additional file 1: Fig. S1.** The pulse sequence diagram for respiratory navigated Look-Locker Imaging (NALLI). **Table S1.** Cardiac function according to disease duration. **Table S2.** Myocardial native T1. **Table S3.** Myocardial extracellular volume fractions (ECV). **Table S4.** Results of the normal control study

## Data Availability

The datasets used and/or analyzed during the current study are available from the corresponding author on reasonable request.

## Abbreviations

CMR: Cardiovascular magnetic resonance
ECV: Extracellular volume fraction
ECG Electrocardiogram EDVI: End-diastolic volume index
EF: Ejection fraction
LGE: Late gadolinium enhancement
LV: Left ventricle/left ventricular
PH: Pulmonary hypertension
ROI: Region of interest
RV: Right ventricle/right ventricular
SIP: Septal insertion point
