# Supplementary material for: Serial T1 mapping of right ventricle in pulmonary hypertension: comparison with histology in an animal study
Source: J Cardiovasc Magn Reson. 2021 May 27;23:64. doi: 10.1186/s12968-021-00755-y (PMC8157452; doi:10.1186/s12968-021-00755-y)
Supplement: Supplementary file 1 — Additional file 1: Fig. S1. The pulse sequence diagram for respiratory navigated Look-Locker Imaging (NALLI). Table S1. Cardiac function according to disease duration. Table S2. Myocardial native T1. Table S3. Myocardial extracellular volume fractions (ECV). Table S4. Results of the normal control study [file 12968_2021_755_MOESM1_ESM.docx]

**Supplemental Materials**

**Figure legends**

**Supplemental Figure 1.** The pulse sequence diagram for respiratory navigated Look-Locker Imaging (NALLI).

Inversion recovery (IR)-weighted images are acquired during the acquisition duration (AD) in the same manner as prospectively electrocardiogram (ECG)-gated cine imaging. The relaxation duration (RD) is the quiescent time until the full recovery of the net magnetization toward equilibrium. At the beginning of the AD, the IR pulse drives the inversion of the net magnetization, and then cine imaging is performed in a multi-slice manner. The navigation scans are performed by measuring the center of the k-space to the detected motion-corrupted cardiac cycles. The IR-weighted echoes at the motion-corrupted cardiac cycle are replaced by the average of echoes at the adjacent no motion-corrupted cardiac cycles to reduce respiratory motion artefacts. To calculate T1 maps, an identical cardiac phase is used (with the IR-weighted images used for the T1 map of the 3rd cardiac phase indicated by the red dashed lines in the diagram) for all heart beats during T1 recovery (in other words, the AD in the pulse sequence diagram), and each inversion time (TI) corresponds to the equation shown as follows: TI = TR x (nCP) + (nHB – 1) x RRI, where, TR is the repetition time, nCP is the number of cardiac phases, nHB is the number of heart beats, and RRI is the averaged RR interval. Next, T1 maps are obtained by applying a three-parameter non-linear least square fit on a pixel-by-pixel basis as is done for the standard Look-Locker pulse sequence and then the Look-Locker correction is applied.

**
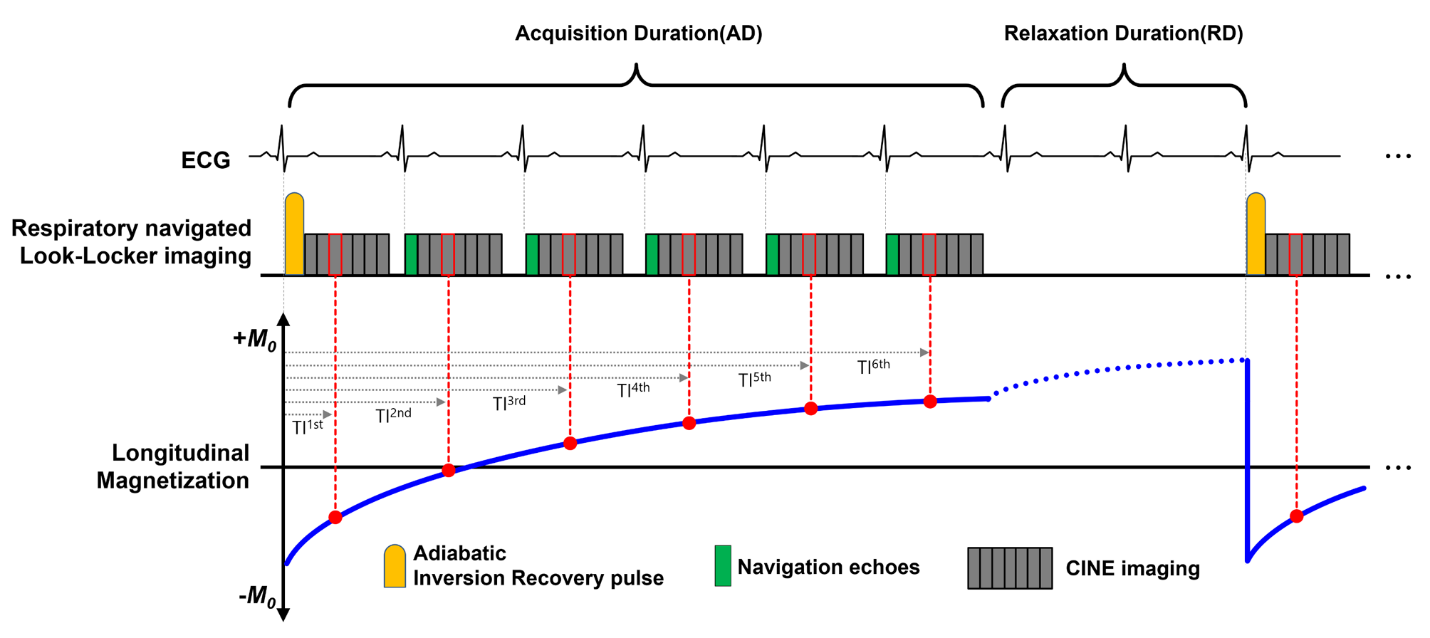
**

**Tables**

**Supplemental Table 1: Cardiac function according to disease duration**

|  | Baseline | 2 weeks | 4 weeks | 6 weeks | 8 weeks |
| --- | --- | --- | --- | --- | --- |
| RVEDVI (ml/m^2^) | 10.1 ± 0.4 | 10.3 ± 0.3 | 11.7 ± 0.7 | 12.3 ± 0.3 | 13.2 ± 0.3 |
| *P* value* |  | 0.262 | 0.004 | 0.004 | 0.004 |
| *P* value† |  | 0.262 | 0.004 | 0.109 | 0.004 |
| LVEDVI (ml/m^2^) | 10.4 ± 0.9 | 10.4 ± 0.5 | 9.7 ± 0.4 | 9.1 ± 0.4 | 8.7 ± 0.5 |
| *P* value* |  | > 0.999 | 0.225 | 0.004 | 0.009 |
| *P* value† |  | > 0.999 | 0.015 | 0.009 | 0.180 |
| RVEF (%) | 67.6 ± 2.0 | 64.9 ± 4.1 | 53.4 ± 4.4 | 49.9 ± 3.2 | 43.5 ± 4.1 |
| *P* value* |  | 0.261 | 0.004 | 0.004 | 0.004 |
| *P* value† |  | 0.261 | 0.004 | 0.150 | 0.025 |
| LVEF (%) | 66.3 ± 2.2 | 65.6 ± 1.5 | 61.2 ± 4.0 | 56.4 ± 4.0 | 50.6 ± 4.4 |
| *P* value* |  | 0.624 | 0.037 | 0.004 | 0.004 |
| *P* value† |  | 0.624 | 0.090 | 0.055 | 0.030 |

Data are means ± standard deviations.

* Between the baseline group and each duration group.

† Between two sequentially adjacent duration groups.

LVEDVI, left ventricular end-diastolic volume index; LVEF, left ventricular ejection fraction; RVEDVI, right ventricular end-diastolic volume index; RVEF, right ventricular ejection fraction

**Supplemental Table 2: Myocardial native T1**

|  | Baseline | 2 weeks | 4 weeks | 6 weeks | 8 weeks |
| --- | --- | --- | --- | --- | --- |
| RV (ms) | 1541 ± 33 | 1557 ± 38 | 1581 ± 32 | 1640 ± 41 | 1746 ± 39 |
| *P* value* |  | 0.699 | 0.093 | 0.004 | 0.002 |
| *P* value† |  | 0.699 | 0.394 | 0.026 | 0.004 |
| Septum (ms) | 1552 ± 39 | 1568 ± 30 | 1591 ± 29 | 1613 ± 31 | 1644 ± 39 |
| *P* value* |  | 0.485 | 0.132 | 0.022 | 0.009 |
| *P* value† |  | 0.485 | 0.240 | 0.162 | 0.223 |
| Anterior SIP (ms) | 1562 ± 42 | 1571 ± 44 | 1623 ± 30 | 1650 ± 32 | 1705 ± 83 |
| *P* value* |  | 0.563 | 0.026 | 0.004 | 0.002 |
| *P* value† |  | 0.563 | 0.058 | 0.180 | 0.240 |
| Inferior SIP (ms) | 1529 ± 42 | 1578 ± 31 | 1668 ± 48 | 1721 ± 61 | 1760 ± 50 |
| *P* value* |  | 0.093 | 0.002 | 0.002 | 0.002 |
| *P* value† |  | 0.093 | 0.002 | 0.132 | 0.310 |
| LV (ms) ‡ | 1576 ± 37 | 1560 ± 36 | 1549 ± 19 | 1565 ± 33 | 1600 ± 48 |

Data are means ± standard deviations.

* Between the baseline group and each duration group.

† Between two sequentially adjacent duration groups.

‡ No significant difference seen with the Kruskall-Wallis test (*P =* 0.349).

LV, left ventricle; RV, right ventricle; SIP, septal insertion point

**Supplemental Table 3: Myocardial extracellular volume fractions (ECV)**

|  | Baseline | 2 weeks | 4 weeks | 6 weeks | 8 weeks |
| --- | --- | --- | --- | --- | --- |
| RV (%) | 17.2 ± 1.3 | 17.53 ± 0.74 | 18.6 ± 1.6 | 22.6 ± 1.7 | 25.7 ± 1.3 |
| *P* value* |  | 0.699 | 0.132 | 0.002 | 0.002 |
| *P* value† |  | 0.699 | 0.394 | 0.004 | 0.009 |
| Septum (%) | 17.5 ± 2.0 | 18.0 ± 2.3 | 19.6 ± 1.8 | 20.7 ± 1.9 | 21.0 ± 1.6 |
| *P* value* |  | 0.818 | 0.093 | 0.041 | 0.015 |
| *P* value† |  | 0.818 | 0.310 | 0.240 | 0.818 |
| Anterior SIP (%) | 17.9 ± 1.5 | 17.4 ± 1.5 | 21.3 ± 2.5 | 22.4 ± 2.1 | 23.8 ± 3.5 |
| *P* value* |  | 0.485 | 0.015 | 0.004 | 0.002 |
| *P* value† |  | 0.485 | 0.015 | 0.485 | 0.818 |
| Inferior SIP (%) | 17.4 ± 2.0 | 19.9 ± 2.1 | 22.9 ± 1.5 | 24.1 ± 1.6 | 25.5 ± 1.9 |
| *P* value* |  | 0.093 | 0.002 | 0.002 | 0.002 |
| *P* value† |  | 0.093 | 0.015 | 0.240 | 0.180 |
| LV (%)‡ | 17.7 ± 1.1 | 18.1 ± 2.5 | 17.5 ± 1.8 | 19.0 ± 2.0 | 20.1 ± 2.6 |

Data are means ± standard deviations.

* Between the baseline group and each duration group.

† Between two sequentially adjacent duration groups.

‡ No significant difference seen with the Kruskall-Wallis test (*P =* 0.240).

LV, left ventricle; RV, right ventricle; SIP, septal insertion point

**Supplemental Table 4: Results of the normal control study**

|  | Baseline | 4 weeks | 8 weeks | *P* value |
| --- | --- | --- | --- | --- |
| Characteristics |  |  |  |  |
| Body weight (g) | 267.8 ± 8.1 | 369.7 ± 8.7 | 478.3 ± 6.9 | < 0.001 |
| Hematocrit (%) | 53.8 ± 2.0 | 55.9 ± 3.8 | 56.3 ± 1.7 | 0.264 |
| Heart rate (bpm) | 283 ± 13 | 305 ± 13 | 296 ± 16 | 0.076 |
| Cardiac function |  |  |  |  |
| RVEDVI (ml/m2) | 10.1 ± 0.4 | 10.4 ± 0.6 | 10.5 ± 0.4 | 0.244 |
| LVEDVI (ml/m2) | 10.4 ± 0.9 | 10.2 ± 0.4 | 10.9 ± 0.4 | 0.147 |
| RVEF (%) | 67.6 ± 2.0 | 63.5 ± 2.4 | 66.8 ± 4.9 | 0.171 |
| LVEF (%) | 66.3 ± 2.2 | 64.4 ± 5.0 | 65.1 ± 2.8 | 0.580 |
| Native T1 values |  |  |  |  |
| RV (ms) | 1541 ± 33 | 1558 ± 38 | 1573 ± 35 | 0.244 |
| LV (ms) | 1576 ± 37 | 1563 ± 41 | 1592 ± 46 | 0.548 |
| Myocardial ECV |  |  |  |  |
| RV (%) | 17.2 ± 1.3 | 17.9 ± 1.4 | 17.8 ± 0.8 | 0.504 |
| LV (%) | 17.7 ± 1.1 | 17.1 ± 1.7 | 18.4 ± 1.4 | 0.291 |
| Collagen density (%) | 4.7 ± 0.5 | 4.9 ± 0.3 | 5.1 ± 0.4 | 0.331 |

Data are means ± standard deviations.

ECV, extracellular volume fraction; LV, left ventricle; LVEDVI, left ventricular end-diastolic volume index; LVEF, left ventricular ejection fraction; RV, right ventricle; RVEDVI, right ventricular end-diastolic volume index; RVEF, right ventricular ejection fraction
